# Human Proteome Microarray identifies autoantibodies to tumor‐associated antigens as serological biomarkers for the diagnosis of hepatocellular carcinoma

**DOI:** 10.1002/1878-0261.13371

**Published:** 2023-01-21

**Authors:** Qian Yang, Hua Ye, Guiying Sun, Keyan Wang, Liping Dai, Cuipeng Qiu, Jianxiang Shi, Jicun Zhu, Xiao Wang, Peng Wang

**Affiliations:** ^1^ The State Key Laboratory of Esophageal Cancer Prevention & Treatment Zhengzhou University China; ^2^ Department of Prenatal Diagnosis Center The Third Affiliated Hospital of Zhengzhou University China; ^3^ Department of Epidemiology and Health Statistics and Henan Key Laboratory of Tumor Epidemiology, College of Public Health Zhengzhou University China; ^4^ Henan Institute of Medical and Pharmaceutical Sciences Zhengzhou University China

**Keywords:** biomarker, diagnosis, hepatocellular carcinoma, Human Proteome Microarray

## Abstract

The identification of the high‐efficiency and non‐invasive biomarkers for hepatocellular carcinoma (HCC) detection is urgently needed. This study aims to screen out potential autoantibodies to tumor‐associated antigens (TAAbs) and to assess their diagnostic value for HCC. Fifteen potential TAAbs were screened out from the Human Proteome Microarray by 30 HCC sera and 22 normal control sera, of which eight passed multiple‐stage validations by ELISA with a total of 1625 human serum samples from normal controls (NCs) and patients with HCC, liver cirrhosis, chronic hepatitis B, gastric cancer, esophageal cancer, and colorectal cancer. Finally, an immunodiagnostic model including six TAAbs (RAD23A, CAST, RUNX1T1, PAIP1, SARS, PRKCZ) was constructed by logistic regression, and yielded the area under curve (AUC) of 0.835 and 0.788 in training and validation sets, respectively. The serial serum samples from HCC model mice were tested to explore the change in TAAbs during HCC formation, and an increasing level of autoantibodies was observed. In conclusion, the panel of six TAAbs can provide potential value for HCC detection, and the strategy to identify novel serological biomarkers can also provide new clues in understanding immunodiagnostic biomarkers.

## Graphical abstract

Using the Human Proteome Microarray, the authors screened autoantibodies to tumor‐associated antigens (TAAbs) to identify serological biomarkers to detect hepatocellular carcinoma (HCC). The diagnostic value for HCC of these TAAbs was determined with multiple‐stage validations.

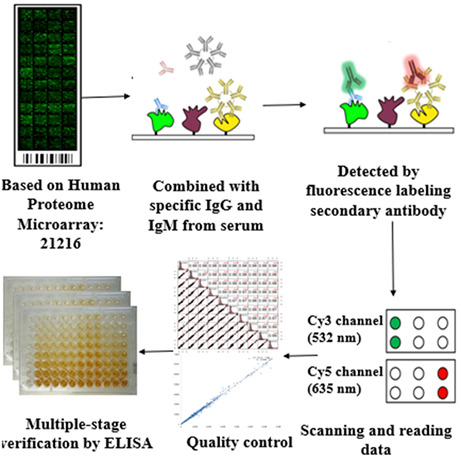

**Abbreviations**
AFP: Alpha‐fetoprotein
AUC: area under the receiver operating characteristic curve
CLD: chronic liver diseases
ELISA: the enzyme‐linked immunosorbent assays
ESCC: esophageal squamous cell cancer
GC: gastric cancer
HCC: hepatocellular carcinoma
NC: normal controls
PP: predictive probability value
ROC curve: receiver operator characteristic curve
SD: standard deviation
Se: sensitivity
Sp: specificity
TAAbs: autoantibodies against tumor‐associated antigens
TAAs: tumor‐associated antigens

## 1 Introduction

Hepatocellular carcinoma (HCC), as the main liver cancer, remains a substantial public challenge globally. According to the Global Cancer Statistics 2020, HCC ranked the fifth diagnosed cancer and the second leading cause of cancer‐related death for both male and female worldwide [1]. Same as other cancers, the five‐year survival rate should be greatly improved if a patient with HCC can be diagnosed at early stage [2]. But, only < 50% of HCC can be diagnosed at early stage due to the lack of reliable noninvasive screening tests in the at‐risk individuals with chronic liver diseases [2, 3, 4, 5, 6]. Therefore, it is urgent for us to find more novel biomarkers for early diagnosis of HCC.

Many studies have indicated that autoantibodies against tumor‐associated antigens (TAAbs) can be considered as sensitive immune sensors in tumorigenesis. Due to the nature of early appearance, stable existence, and easy measurement in sera, TAAbs can be used as serological biomarkers for early detection of cancers [7, 8, 9]. Previous studies from our laboratory and others showed that TAAbs might play a role in the development of HCC because autoantibodies appeared very early, and the elevated autoantibodies could be associated with malignant transition to HCC [10, 11, 12]. So far, many studies were mainly focused on three forms of TAAs (mutated proteins, abnormal expression proteins, and other posttranslationally modified proteins) [13]. Also, the studies about TAAbs as biomarkers for HCC detection were mainly derived from scattered reports or proteins encoded by certain genes [10]. Thus, there are not many reports about the research to screen novel anti‐TAA autoantibodies from Human Proteome Microarray for early detection of HCC.

Protein microarray technology was widely used to analyze autoantibodies in previous studies by our laboratory [14] or others [15, 16]. It could rapidly and thoroughly screen the whole proteome to identify TAAbs in human serum samples [17]. The development of HCC is closely associated with the existence of the at‐risk liver diseases, such as chronic hepatitis B (CHB) and liver cirrhosis (LC) [18, 19]. Therefore, exploring the appearance and change of anti‐TAA autoantibodies in patients at different stages of HCC development would help to find more valuable biomarkers for the early detection of HCC. Here, we designed a large‐scale multistage study to identify the potential TAAbs, and developed an immunodiagnostic model for HCC detection, which could discriminate AFP‐negative HCC patients from normal control (NC), and also early‐stage HCC patients from NC, CHB control, the at‐risk control, LC control, and all non‐HCC control. Moreover, the serial sera from HCC model mice were also tested to further confirm the possibility of TAAbs in the panel as biomarkers for early detection of HCC.

## 2 Materials and methods

### 2.1 Human serum samples

All serum samples were from the sera bank of Tumor Epidemiology of Laboratory of Zhengzhou University (Henan, China). Thirty HCC serum samples in 10 serum pools (three sera for one pool) and 18 normal control serum samples in six serum pools (three sera for one pool) and four individual sera were used to screen candidate antigens in Human Proteome Microarrays. Five independent sets successively comprised a test set (80 HCC sera and 80 NC sera), a training set (220 HCC sera and 220 NC sera), a validation set (160 HCC sera and 160 NC sera), an at‐risk set (157 LC sera and 96 CHB sera), and a specific validation set (80 GC sera and 120 ESCC sera, and 66 CRC sera).

The detailed characteristics of all participants by ELISA were shown in Table 1. All patients were diagnosed according to the Chinese Guidelines for the liver diseases including by the Chinese Society of Hepatology [20, 21, 22]. Namely, the diagnosis of hepatocellular carcinoma or liver cirrhosis is on the basis of at least two imaging methods (CT, MRI, and ultrasound), biochemistry (AFP or AFP‐L3), histopathology of liver biopsy samples by clinical physicians and pathologists. Patients with chronic HBV infection referred to the people caused by persistent HBV infection for the last 6 months. The exclusion criteria of the serum samples from the patients and normal controls were as follows respectively: (a) The HCC patients have received anticancer treatment such as radiotherapy or chemotherapy before collecting the serum samples. (b) The HCC patient had a history of other solid tumors. (c) The normal control had the history of hepatic diseases, autoimmune diseases, alcoholism, or abnormal liver biochemistry. All human participants have signed informed consent and the study was approved by the Institutional Review Board of Zhengzhou University (ZZURIB2019001) and conformed to the standards set by the Declaration of Helsinki.

**Table 1:** The demographic and clinical characteristics of the human participants for the ELISA. At‐risk indicates non‐cancerous carcinoma diseases, including chronic hepatitis B and liver cirrhosis. AFP, alpha‐fetoprotein; CHB, chronic hepatitis B; CRC, colorectal cancer; ESCC, esophageal cancer; GC, gastric cancer; HCC, hepatocellular carcinoma; IA, inapplicable; NA, not available; NC, normal control; SD, standard deviation.

Characteristics	Test set		Training set			Validation set			Specific validation set			
	HCC, *n* (%)	NC, *n* (%)	HCC, *n* (%)	NC, *n* (%)	HCC, *n* (%)	NC, *n* (%)	Cirrhosis, *n* (%)	CHB, *n* (%)	GC, *n* (%)	ESCC, *n* (%)	CRC, *n* (%)	NC, *n* (%)
Sample size	80	80	220	220	160	160	157	96	80	120	66	186
Gender, *n* (%)												
Male	56 (70.0)	56 (70.0)	175 (79.5)	175 (79.5)	127 (79.4)	127 (79.4)	125 (79.6)	79 (82.3)	59 (74.2)	89 (74.2)	32 (48.5)	121 (65.1)
Female	24 (30.0)	24 (30.0)	45 (20.5)	45 (20.5)	33 (20.6)	33 (20.6)	32 (20.4)	17 (17.7)	21 (25.8)	31 (25.8)	34 (51.5)	31 (34.9)
Age												
Mean ± SD	54.0 ± 10.6	54.0 ± 10.5	54.0 ± 11.4	54.0 ± 11.2	53.9 ± 11.8	53.9 ± 11.7	54.0 ± 9.8	51.0 ± 9.0	64.2 ± 9.2	64.4 ± 9.7	59.7 ± 30.5	61.7 ± 16.1
Age range	26–79	25–78	20–83	21–82	20–82	21–83	20–79	23–80	40–74	41–87	28–89	28–89
TNM, *n* (%)												
I + II	48 (60.0)	IA	99 (45.0)	IA	70 (43.8)	IA	IA	IA	24 (30.0)	20 (16.7)	0 (0.0)	IA
III + IV	32 (40.0)	IA	75 (34.1)	IA	56 (35.0)	IA	IA	IA	17 (21.3)	23 (19.2)	0 (0.0)	IA
NA	0 (0.0)	IA	46 (20.9)	IA	34 (21.2)	IA	IA	IA	39 (48.7)	77 (64.2)	66 (100.0)	IA
AFP, *n* (%)												
≥ 20 ng·mL^−1^	55 (68.7)	0 (0.0)	118 (53.6)	3 (0.1)	87 (54.4)	2 (1.3)	39 (24.8)	26 (27.1)	0 (0.0)	0 (0.0)	0 (0.0)	0 (0.0)
< 20 ng·mL^−1^	25 (31.3)	80 (100.0)	84 (38.2)	85 (39.1)	67 (41.9)	85 (53.1)	63 (40.1)	42 (43.8)	0 (0.0)	0 (0.0)	0 (0.0)	0 (0.0)
NA	0 (0.0)	0 (0.0)	18 (8.2)	132 (60.0)	6 (3.7)	73 (45.6)	55 (35.0)	28 (29.2)	80 (100.0)	120 (100.0)	66 (100.0)	186 (100.0)
CHB, *n* (%)												
No	22 (27.5)	80 (100.0)	56 (25.5)	220 (100.0)	33 (20.6)	1 60 (100.0)	43 (27.4)	0 (0.0)	0 (0.0)	0 (0.0)	0 (0.0)	0 (0.0)
Yes	58 (72.5)	0 (0.0)	133 (60.5)	0 (0.0)	109 (68.1)	0 (0.0)	65 (41.4)	96 (100.0)	0 (0.0)	0 (0.0)	0 (0.0)	0 (0.0)
NA	0 (0)	0 (0.0)	31 (14.1)	0 (0.0)	18 (11.3)	0 (0.0)	49 (312)	0 (0.0)	80 (100.0)	120 (100.0)	66 (100.0)	186 (100.0)

### 2.2 Mouse serum samples

Mouse serial sera were from primary HCC model mice housed in SPF conditions, which were established by hydrodynamic high‐pressure transfection technology. Ten male wild‐type C57BL/6J mice at the age of 6–8 weeks purchased from Shanghai Nanfang Model Biotechnology Co., Ltd were equally divided into two groups (HCC group and control group) in this experiment. The mixed ratio of the five plasmids (pCMV/SB, pT3‐EF1a‐c‐met, pT3‐N90‐β‐catenin, lentiCRISPR‐sgPTEN, lentiCRISPR‐sgp53) were 1 : 1 : 1 : 1 : 1 in this study. Then, the mixed plasmids were dissolved in physiological saline equivalent to 8–12% of the body weight of the mice. Finally, the above‐mixed solution was injected into the tail vein of the HCC group mice by hydrodynamic high‐pressure transfection technology at a speed of < 5 s. The mice in the control group were cotransfected with five corresponding empty vectors. The primary HCC mouse models were formed in 6 weeks after cotransfection. The mouse sera were collected in the second week, the fourth week, and the sixth week in the process of hepatocarcinogenesis, respectively. The hepatic tissues of the mice were removed for the pathological diagnosis under deep anesthesia when the malignant lesions were initially formed. The animal study was reviewed and approved by Experimental Animal Ethics Committee of the Academy of Military Medical Sciences 2020‐680.

### 2.3 The Human Proteome Microarray

The 21 216 proteins included in Human Proteome Microarrays were purchased from CDI labs (http://cdi.bio/huprot). The Human Proteome Microarrays used in this study were made by BC biotechnology Co., LTD (Guangzhou, China) and were used to screen candidate biomarkers in 30 HCC serum samples in 10 sera pools (three samples matched by age and gender were mixed together and used as one pool). Same as HCC sera, six sera pools from 18 normal control serum samples were made (every three samples matched by age and gender). In addition, four individual normal human sera were also used.

The experimental principles about the protein microarray were described in our previous study [10]. Duplicate spots were set for each protein. Besides, the positive and the negative controls were set for the quality control. The experimental procedures were the same as a reported study for gastric cancer [23].

### 2.4 Recombinant proteins and the detection of TAAbs by ELISA

All 15 proteins used in ELISA were purchased from Cloud‐clone Corporation (PRKCZ and DUSP6, Wuhan, China) or CUSABIO (SF3B3, RUNX1T1, SARS, PAIP1, CAST, MAGEA12, CCDC6, RAD23A, NOL7, CRLF3, NAP1L4, SH2B1, LARP6, Wuhan, China). The SDS/PAGE gel was run to confirm the concentration, purity, and molecular weight of each protein before use.

The sera for ELISA in this study were stored at −80 °C. ELISA was used for the detection of the TAAbs level in all four‐stage validation sets. All the proteins for ELISA in this study were individually diluted at appropriate concentrations. The coating concentrations were 0.25 μg·mL^−1^ for PAIP1, NOL7, LARP6, SARS, NAP1L4, CRLF3, CAST and 0.125 μg·mL^−1^ for SF3B3, RUNX1T1, MAGEA12, CCDC6, RAD23A, SH2B1, DUSP6, SH2B1. The detecting agent in this study was the solution of 3, 3′, 5, 5′‐ tetramethyl benzidine (TMB)‐H_2_O_2−_urea. Meanwhile, the stop solution was the sulfuric acid. The HCC, LC, CHB, and NC samples were distributed on each plate. Three blanks were set on each plate for the quality control inside a plate. The five parallel sera were set on each plate for normalization across different plates.

### 2.5 Statistical analysis

The values of each spot in the Human Proteome Microarray analysis, such as the median values of *F*_ij_ (foreground) and *B*_ij_ (background), were extracted by the software of Genepix Pro6.0. The signal‐to‐noise ratio (SNR), which is defined as the ratio of the mean value of *F*_ij_ and *B*_ij_ intensity of each protein, was used for the following analysis in terms of Human Proteome Microarray. *Z*‐score and median normalization were used for differential expression analysis in the Human Proteome Microarray.

The optical density (OD) values obtained by ELISA were compared by Mann–Whitney *U* test between two groups, and the Kruskal‐Wallis *H* test was used to compare the difference among three or four groups. Receiver operating characteristics curve (ROC) was generated to assess the diagnostic performance with the sensitivity, specificity, and the area under ROC (AUC) with 95% confidence interval (CI) for each of single TAAbs. To establish the immunodiagnostic models of the TAAbs either alone, or combination with AFP, between HCC patients and non‐HCC patients' controls, the non‐conditional logistic regression was used in the training set. Then we used another independent validation set (160 HCC and 160 NC) for the external validation to further explore the performance of the established immunodiagnostic model. ROC analysis in predicted probability (PP) of the model was performed, too. The method of DeLong et al. was used to compare the difference between two ROC curves. *P* < 0.05 was considered to be significant difference by two sides. All data were analyzed by spss software (version 26.0), rstudio (version 3.6.1), or graphpad prism software (version 5.0).

## 3 Results

### 3.1 Overall study design

**Fig. 1:**
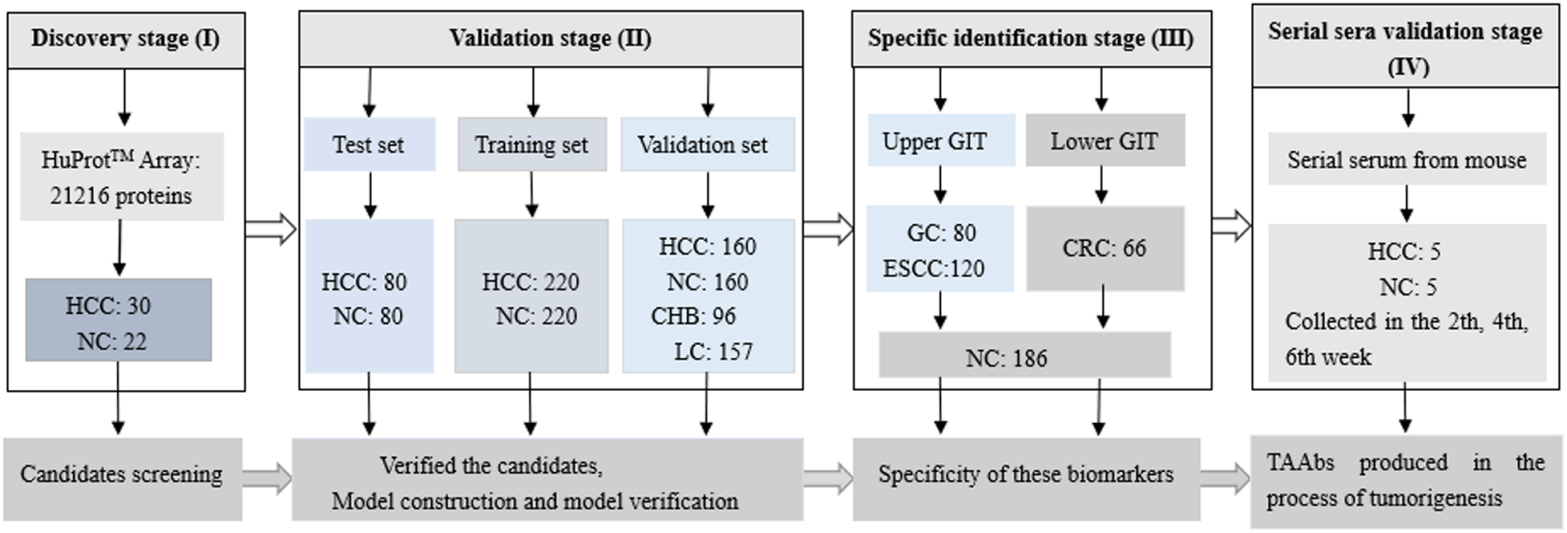
Flow chart of the study design. CHB, chronic hepatitis B; CRC, colorectal cancer; ESCC, esophageal cancer; GC, gastric cancer; GIT, gastrointestinal tumor; HCC, hepatocellular carcinoma; NC, normal controls.

The study included four stages (Fig. 1): discovery stage (I), validation stage (II), specific validation stage (III), and serial sera validation stage (IV). In discovery stage (I), 10 HCC sera pools, and six normal control sera pools and four individual normal control samples were used to screen candidate TAAs on Human Proteome Microarray. To screen the best candidate biomarkers, four criteria were set: (a) *P* < 0.05 by Mann–Whitney U test and the SNR were higher in the HCC group than that in the NC group. (b) Fold change ≥ 1.5. (c) The positive rate ≥ 60% in the HCC group. (d) The positive rate ≤ 10% in the NC group. According to the criteria, 25 candidate biomarkers were identified. Finally, 15 candidate biomarkers (autoantibodies to RUNX1T1, RAD23A, CAST, PRKCZ, SF3B3, SARS, DUSP6, PAIP1, SH2B1, NAP1L4, CRLF3, LARP6, NOL7, MAGEA12, and CCDC6) were screened out based on the cancer literature and the database. In validation stage (II), a test set, including 160 serum samples (80 HCC sera and 80 NC sera matched to HCC patients by age and gender), was employed for preliminary verification of the 15 candidate TAAbs, and nine differentially expressed TAAbs were confirmed. Then, two independent sets, namely training set and validation set, comprised of 1013 serum samples from 380 HCC patients, 380 healthy controls, 96 CHB patients, and 157 LC patients were applied for further validation of the differentially expressed TAAbs, and the construction and verification of an immunodiagnostic model. In specific validation stage (III), all serum samples from 80 gastric cancer (GC) patients, 120 esophageal cancer (ESCC) patients, 66 colorectal cancer (CRC) patients, and 186 normal controls matched to cancer patients by age and gender in proportion were used to test the specificity of the eight identified TAAbs. In serial sera validation stage (IV), 30 serial sera from primary HCC model mice and control model mice were tested to further validate the dynamic changes of the identified TAAbs during hepatocarcinogenesis.

### 3.2 Characteristics of the human population

The demographic and clinical characteristics of the human participants for ELISA were shown in Table 1. There was no significant statistical difference in the distribution of age and gender between patient group and normal control group in each set. The positive rate of AFP was more than 50% for HCC patients in each set, while it was 24.8% and 27.1% respectively for patients with liver cirrhosis and chronic hepatitis B. The distributions of the TNM stage and individuals with AFP‐positive of each HCC group in training set and validation set were compared by Pearson's chi‐squared test, and no statistical significance was found.

### 3.3 Serum autoantibodies in discovery stage (I)

In discovery stage (I), based on a series of criteria (*P* < 0.05, fold change ≥ 2, the sensitivity ≥ 60%, the specificity ≥ 90%, the Youden index ≥ 50%, and the significant elevation of the value of SNR in HCC group compared to that in NC group) by the intersection of the calculation methods (*Z*‐score and median normalization), 15 candidate TAAs (RUNX1T1, RAD23A, CAST, PRKCZ, SF3B3, SARS, DUSP6, PAIP1, SH2B1, NAP1L4, CRLF3, LARP6, NOL7, MAGEA12, and CCDC6) were screened out from Human Proteome Microarray. The basic information of the 15 candidate TAAs was showed in Table S1.

### 3.4 Serum autoantibodies in validation stage (II)

In the validation stage (II) including three sets (test set, training set, validation set), ELISA was employed to profile 1173 serum samples. First, 15 recombinant proteins were used as antigens to detect the corresponding TAAbs in test set (80 HCC and 80 normal controls). The scatter plots for the level of 15 candidate TAAbs in sera were shown in Fig. S1a. Among these 15 TAAbs, the levels of nine TAAbs (RUNX1T1, RAD23A, CAST, PRKCZ, SF3B3, SARS, DUSP6, PAIP1, and CRLF3) exhibited significantly higher in the HCC patients than that in the NC.

To evaluate the distinguishing ability of these TAAbs more accurately, more samples were enrolled in the training and validation set. In the training set (220 HCC and 220 NC), the levels of these nine TAAbs were significantly higher in HCC patients than those in NC, except anti‐CRLF3 (Figs S1b and S2a). Based on the analysis of eight differentially expressed TAAbs, anti‐PAIP1 and anti‐PRKCZ showed higher AUC of 0.705 and 0.701, respectively. In the validation set (160 HCC and 160 NC), as shown in Figs S1c and S2b, anti‐RAD23A showed the highest AUC of 0.685, with a sensitivity of 28.5% and a specificity of 90.0%. Anti‐PAIP1 and anti‐PRKCZ still showed higher AUC of 0.661 and 0.663. AUCs of other differentially expressed anti‐TAA (RUNX1T1, CAST, SF3B3, SARS, and DUSP6) successively were 0.594, 0.625, 0.646, 0.611, and 0.610 (*P* < 0.05). The levels of these eight TAAbs gave similar trends in training and validation sets, and the results in both sets were consistent with those in test set except anti‐CRLF3 (Fig. S1).

### 3.5 Establishment and validation of the immunodiagnostic model for HCC detection

**Fig. 2:**
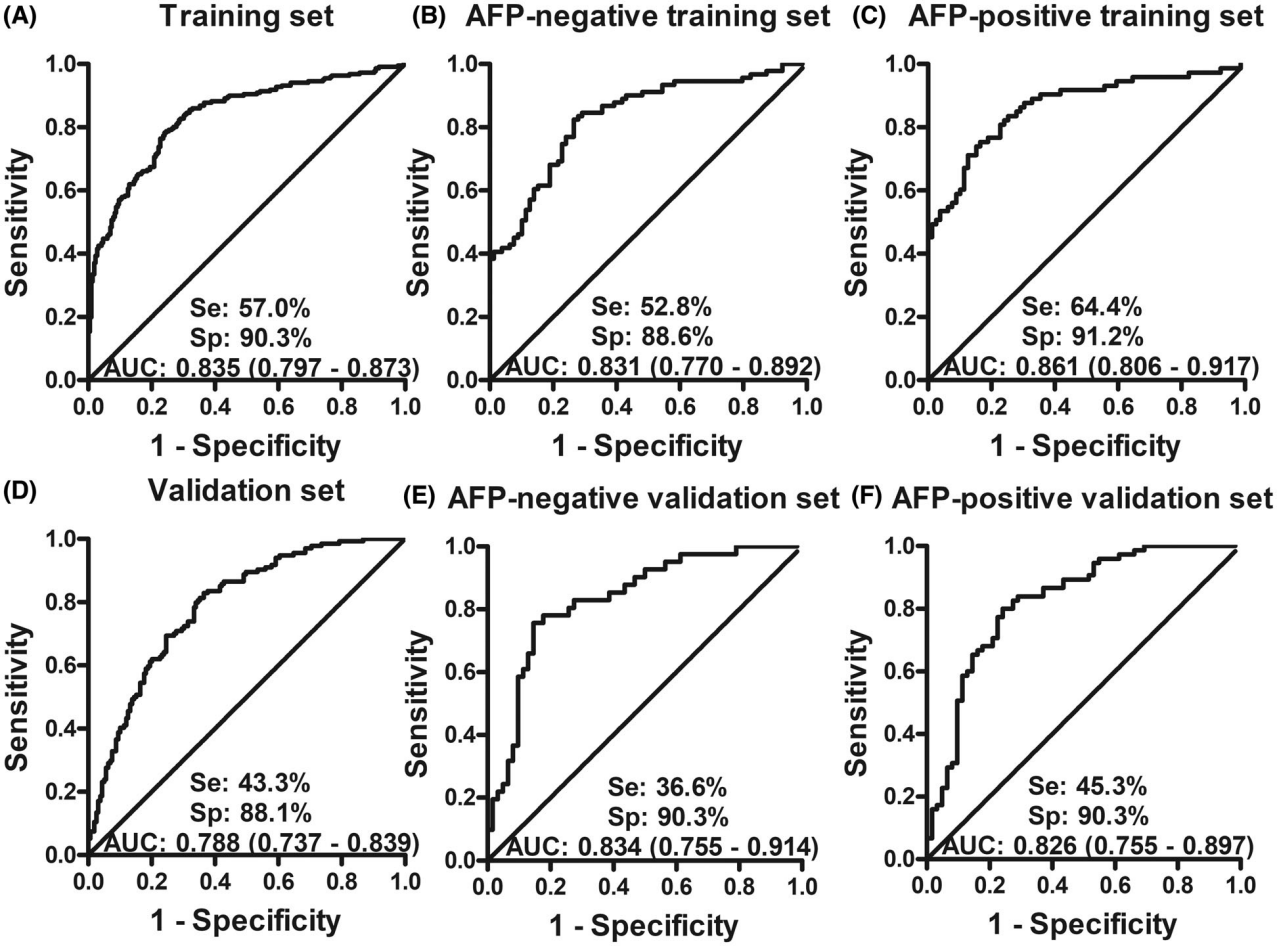
Receiver operator characteristic curves (ROC) of predictive probability (PP) values of the immunodiagnostic model in training set (A), AFP‐negative training set (B), AFP‐positive training set (C), validation set (D), AFP‐negative validation set (E), and AFP‐positive validation set (F). AFP‐negative means the HCC patients with serum AFP concentration lower than 20 ng·mL^−1^, AFP‐positive indicates that the HCC patients with serum AFP concentration not < 20 ng·mL^−1^, Se is an abbreviation for “sensitivity”, Sp is the abbreviation of “specificity”, AUC means the area under curve.

Next, the serum samples from 220 HCC and 220 NC in the training set were selected to establish the binary logistic regression model. The dependent variable was based on whether a participant was considered as HCC or not, the independent variables were the OD values of eight differentially expressed TAAbs in HCC and NC. Finally, six TAAbs (RAD23A, CAST, RUNX1T1, PAIP1, SARS, and PRKCZ autoantibodies) were included in the immunodiagnostic model. The equation of the model was as follows: logit (P = HCC) = 1/(1 + EXP (−(3.498 * RAD23A + 5.516 * CAST − 3.571 * RUNX1T1 + 6.210 * PAIP1 − 7.411 * SARS + 14.352 * PRKCZ − 4.219))). ROC analysis was performed according to the predictive probability of the immunodiagnostic model as shown in Fig. 2A. Finally, the model had an AUC of 0.835 to discriminate individuals with HCC from NC with a sensitivity of 57.0%, specificity of 90.3%, accuracy of 77.3%, and a Kappa value in the training set when the cutoff value was 0.66 (Fig. 2A and Table 2).

**Table 2:** Performance of the immunodiagnostic model and AFP to detect HCC in different sets. The cutoff value of the immunodiagnostic model was set at the maximum Youden index when the specificity was > 90%. The cutoff value of AFP was 20 ng·mL^−1^. +LR, positive likelihood ratio; AFP, alpha‐fetoprotein; AUC, area under curve; −LR, negative likelihood ratio; NPV, negative predictive value; PPV, positive predictive value; Se, sensitivity; Sp, specificity; TAAb, autoantibody to tumor‐associated antigen. AFP‐negative means the HCC patients with serum AFP concentration lower than 20 ng·mL^−1^, AFP‐positive indicates that the HCC patients with serum AFP concentration not < 20 ng·mL^−1^, PP refers the prediction probability (PP) value of the 6‐TAAb panel by the binary logistic regression.

	AUC (95%CI)	Se (%)	Sp (%)	Youden index	+LR	−LR	Accuracy (%)	Kappa	PPV (%)	NPV (%)
Training set										
AFP	0.702 (0.640–0.759)	44.5	95.2	0.397	9.35	0.58	70.0	0.437	98.6	58.3
Model^a^	0.835 (0.797–0.873)	57.0	90.3	0.473	5.86	0.48	73.3	0.469	85.7	67.2
Model + AFP	0.923 (0.886–0.951)	75.3	98.7	0.740	59.45	0.25	81.9	0.623	99.3	60.9
Model in AFP‐negative group	0.831 (0.770–0.892)	52.8	88.6	0.414	4.63	0.53	69.4	0.402	84.2	61.9
Model in AFP‐positive group	0.861 (0.806–0.917)	64.4	91.2	0.556	7.35	0.39	77.6	0.548	79.7	82.8
Validation set										
AFP	0.739 (0.663–0.805)	64.7	96.1	0.631	8.55	0.36	72.0	0.466	98.7	60.2
Model	0.788 (0.737–0.839)	43.3	88.1	0.314	3.62	0.64	67.2	0.317	75.3	64.8
Model + AFP	0.927 (0.890–0.964)	82.8	90.3	0.731	40.72	0.19	85.4	0.694	94.1	73.7
Model in AFP‐negative group	0.834 (0.755–0.914)	36.6	90.3	0.269	4.03	0.68	68.9	0.293	72.7	69.1
Model in AFP‐positive group	0.826 (0.755–0.897)	45.3	90.3	0.356	4.68	0.61	65.7	0.340	85.0	57.7

a The model in this table is the immunodiagnostic model established by the binary logistic regression.

The diagnostic performance of the 6‐TAAb panel was then evaluated by another independent validation set including 160 HCC and 160 NC. As shown in Fig. 2D and Table 2, the differentiation of HCC and NC in the validation set had an AUC of 0.788, a sensitivity of 43.3%, a specificity of 88.1%, and an accuracy of 67.2%, when the cutoff values were set as the maximum Youden index with the specificity ≥ 90%.

### 3.6 The 6‐TAAb panel and AFP in distinguishing HCC from NC

The serum alpha fetoprotein (AFP) was tested by conventional assays (radioimmunoassay). According to the investigators' recommendation for HCC detection [24], alpha‐fetoprotein (AFP) threshold of 20 ng·mL^−1^ was used for dividing HCC patients into AFP‐positive group and AFP‐negative group. For AFP‐negative HCC detection in the training and validation sets, the immunodiagnostic model of the six TAAbs provided sensitivity of 52.8% and 36.6%, specificity of 88.6% and 90.3% with the AUC of 0.831 and 0.834 for the identification of HCC from NC, respectively (Fig. 2B,E). To enhance the diagnostic value for HCC detection, the immunodiagnostic model and AFP were combined in training and validation sets. As shown in Table 2, the combination was able to distinguish HCC from NC with an AUC of 0.923, yielding a sensitivity of 75.3%, a specificity of 98.7%, a Kappa value of 0.623 in the training set. The similar results were observed in the validation set.

### 3.7 The 6‐TAAb panel and AFP in distinguishing HCC from the at‐risk patients

**Fig. 3:**
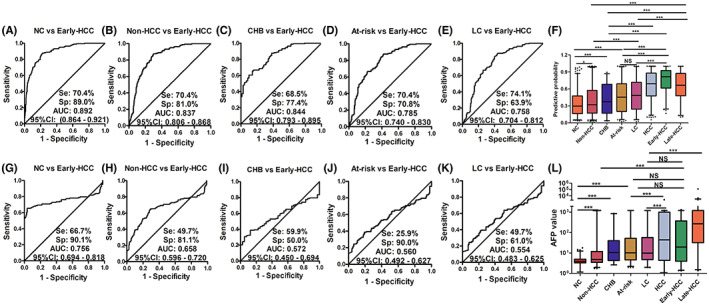
Receiver operator characteristic (ROC) curves and box plots of PP (A–F) and AFP (G–L) in different subgroups. (A–E) show ROC curves of PP for early‐stage HCC versus different controls. (F) Shows the PP levels for HCC patients and different controls. (G–K) shows ROC curves of AFP for early‐stage HCC patients versus different controls. (L) Shows the AFP levels for HCC patients and different controls. PP means predictive probability. AFP means alpha fetoprotein. NC indicates normal controls. At‐risk indicates non‐cancerous carcinoma diseases, including chronic hepatitis B and liver cirrhosis. LC means liver cirrhosis. Non‐HCC indicates non‐hepatocellular carcinoma control, including normal control, patients with chronic hepatitis B, and patients with liver cirrhosis. Early‐HCC indicates patients with early‐stage hepatocellular carcinoma. Late‐HCC indicates patients with late‐stage hepatocellular carcinoma. HCC, hepatocellular carcinoma. Lines on boxes are in the order of 2.5, 25, 50, 75, and 97.5 quantiles from bottom to top. The scatter plots mean the points < 2.5% quantile and more than 97.5% quantile of each group. *: *P* < 0.05, ***: *P* < 0.001, NS: no significant difference, all *P*‐values were calculated by Mann–Whitney *U* test.

The patients with chronic liver diseases are the at‐risk patients with the formation of hepatocellular carcinoma [2]. To further explore the performance of the immunodiagnostic model and AFP in distinguishing HCC from at‐risk patients, we set up an at‐risk control group which included 96 chronic hepatitis B sera and 157 cirrhosis sera, as well as all 380 HCC sera and 380 NC sera from both training and validation sets in this study. As shown in Fig. 3F, the median of the predictive probability of the immunodiagnostic model showed a successively increasing trend with significant differences across six groups as HCC developed (median: 0.297 of normal control, 0.320 of non‐HCC control, 0.373 of CHB control, 0.454 of at‐risk control, 0.484 of LC control, 0.814 of early‐stage HCC group). While, compared with the early‐stage (TNM stage I + II) HCC patients (median: 0.814, mean: 0.924), the PP value tended to decrease a little at the late‐stage (TNM stage III + IV) HCC patients (median: 0.663, mean: 0.873). The levels of PP in patients with late‐stage HCC patients were also significantly higher than those in both the normal control and at‐risk control groups. ROC curves showed the performance of PP level in the diagnosis of early‐stage HCC patients from normal control (AUC of 0.892, sensitivity of 70.4%, specificity of 89.0%), non‐HCC control (AUC of 0.837, sensitivity of 70.4%, specificity of 81.0%), CHB control (AUC of 0.844, sensitivity of 68.5%, specificity of 77.4%), at‐risk control (AUC of 0.785, sensitivity of 70.4%, specificity of 70.8%) and LC control (AUC of 0.758, sensitivity of 74.1%, specificity of 63.9%; Fig. 3A–F).

Interestingly, the plasma AFP concentrations did not show significant difference between CHB control, at‐risk control, or LC control and early‐stage HCC patients (Fig. 3I–L). However, the plasma AFP concentrations in the late‐stage HCC patients showed a remarkable elevated level than those in all different control subgroups. Meanwhile, ROC curves showed that the ability of the AFP to distinguish early‐stage HCC patients from normal controls or non‐HCC controls with AUC of 0.756 or 0.658 was obviously higher than that of AFP to distinguish early‐stage HCC patients from at‐risk controls or LC controls (AUC of 0.560 or 0.564; Fig. 3G,H).

### 3.8 The serum autoantibodies in specific validation stage (III)

**Fig. 4:**
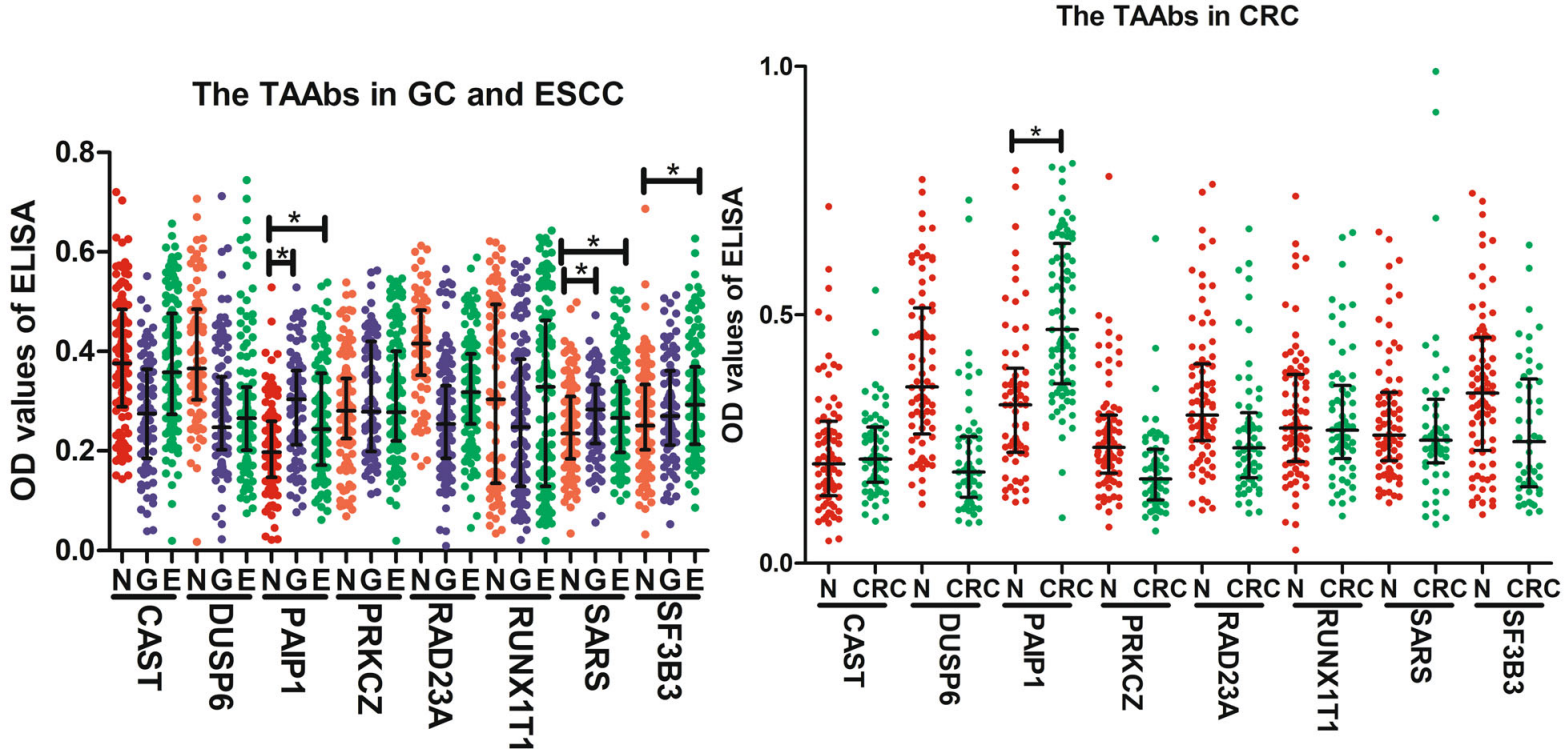
The scatter dot plot of the optical density (OD) values of ELISA for 8 TAAbs in the specific validation set. CRC, colorectal cancer; E, esophageal cancer; G, gastric cancer; N, normal controls. The line at the scatter plots was the median with interquartile range. *P < 0.05 by the Mann–Whitney *U* test. ELISA, enzyme‐linked immunosorbent assay; TAAb, autoantibody to tumor‐associated antigen.

To clarify whether these eight differentially expressed TAAbs identified in HCC patients were specific for HCC detection, we further tested the expression level of these eight TAAbs by ELISA in another specific validation set including other three common gastrointestinal tumors [two common upper gastrointestinal tumors (80 gastric cancer patients, 120 esophageal cancer patients and the matched 120 normal controls), one common lower gastrointestinal tumor (66 colorectal cancer and the matched 66 normal controls)]. As shown in Fig. 4A, the levels of autoantibodies against SARS and PAIP1 were significantly higher in patients with upper gastrointestinal tumors than those in NC (*P* < 0.05). Anti‐SF3B3 autoantibody presented higher level in sera from patients with esophageal cancer than that in the normal controls. Figure 4B showed that only anti‐PAIP1 was significantly higher in sera from patients with colorectal cancer than that in normal control. While, the other five TAAbs, including CAST, DUSP6, PRKCZ, RAD23A, and RUNX1T1, did not show significantly higher levels in sera from patients with the three common gastrointestinal tumors than those in normal control sera. Our results indicated that five of eight identified TAAbs were relatively specific for HCC detection across all four common gastrointestinal cancers.

### 3.9 Titers of TAAbs in serial sera from HCC mouse model mice

**Fig. 5:**
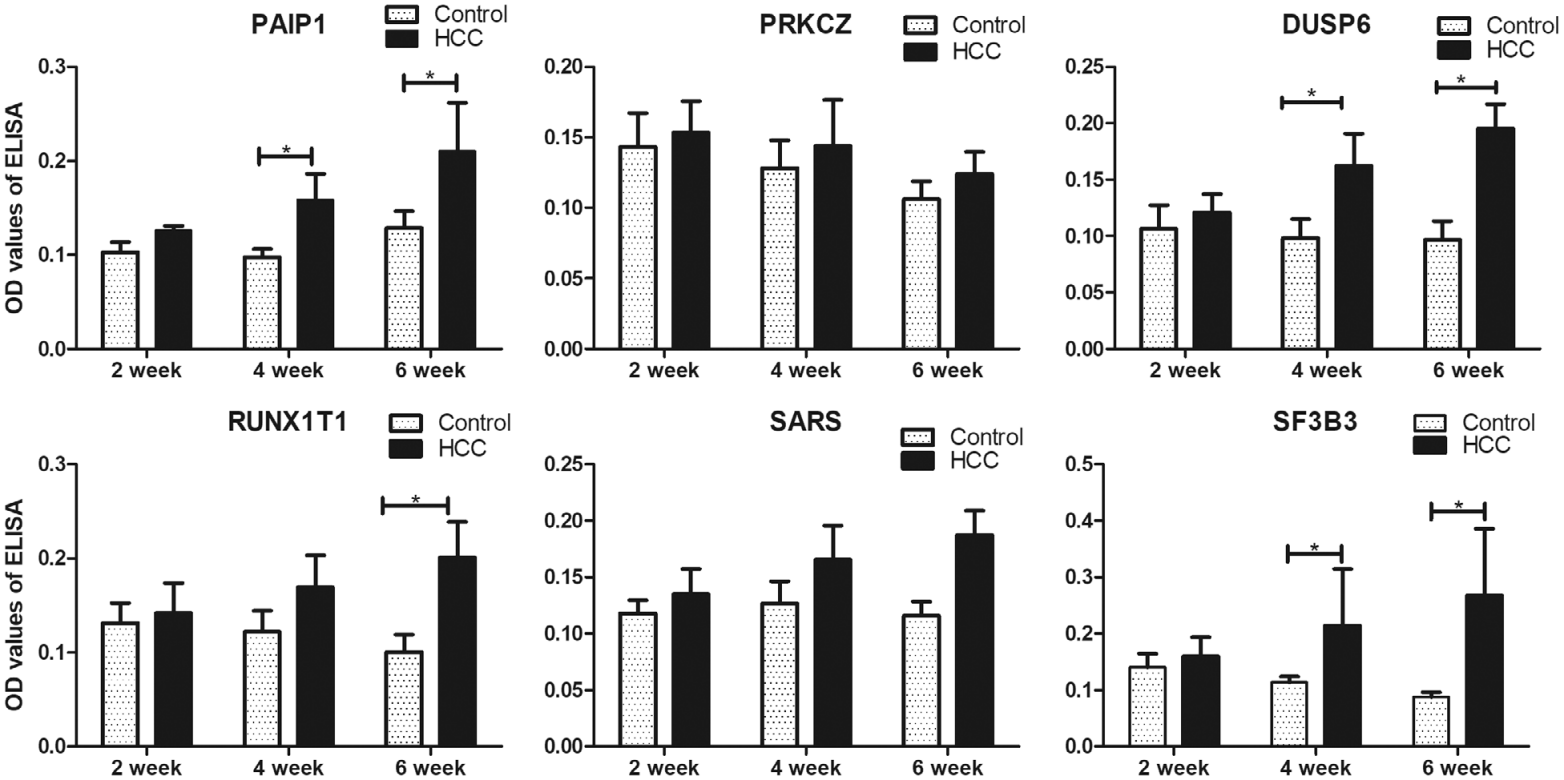
Levels of 6 TAAbs in serial serum samples from the HCC model mice at different time points. HCC, hepatocellular carcinoma. *P < 0.05 by the Kruskal‐Wallis *H* test. The error bars indicate the SEM (standard error). HCC, hepatocellular carcinoma; TAAb, autoantibody to tumor‐associated antigen.

For exploring whether the TAAbs had elevated early before the formation of HCC, the primary HCC mouse models were established on male wild‐type C57BL/6J mice in consideration of the hardship for obtaining the serial sera in the process of hepatocarcinogenesis in human. Five mice with HCC were accounted in the HCC group and five mice with empty vectors were in the control group. A total of 30 serial serum samples were collected from five HCC mice and five control mice in the second week, the fourth week, and the sixth week in the process of hepatocarcinogenesis. Then we used ELISA to test the expression levels of six representative autoantibodies against PAIP1, PRKCZ, DUSP6, RUNX1T1, SF3B3, and SARS. Interestingly, as shown in Fig. 5, all six anti‐TAAs autoantibodies showed an increasing trend with time going, whereas they appeared at relatively stable lower levels in control groups. Especially, the levels of antibodies against PAIP1, DUSP6, and SF3B3 showed a significant raise in the HCC mouse group than in the control group in the fourth week and the sixth week. The results demonstrated that these TAAbs may rise in precancerous lesions of the liver.

## 4 Discussion

Although pathological examination and imaging examination, as the “gold standard”, have been widely used for clinical HCC diagnosis, the serum tumor biomarkers still present an appealing potential for early detection and surveillance of HCC due to the non‐invasive and objective pattern. Up to now, AFP is the only widely used screening biomarker for the clinical practice of the liver cancer by the National Health Commission of the People's Republic of China [25]. However, about 40% of HCC patients showed a negative AFP value and 20% normal people presented a positive AFP value even if the most efficient cutoff is considered [24, 26]. Besides, the at‐risk patients with chronic liver diseases usually present an elevated serum AFP concentration [27]. Therefore, serum autoantibodies against TAAs may be the promising biomarkers for HCC detection owing to its nature of early occurrence and easy detection [15].

This study was divided into discovery stage (I), validation stage (II), specific identification stage (III), and serial sera validation stage (IV) for identifying the TAAbs as biomarkers for HCC detection. Based on the four‐stage design in this study, we finally focused eight autoantibodies against RUNX1T1, RAD23A, CAST, PRKCZ, SF3B3, SARS, DUSP6, and PAIP1 as biomarkers for HCC detection, which were screened out by different experimental techniques and confirmed in a few independent cohorts. Moreover, all of them except anti‐PAIP1, anti‐SARS, and anti‐SF3R3 were specific for HCC detection among common gastrointestinal tumors. Compared with the normal control group, at least two of the three gastrointestinal cancers including gastric cancer group, the esophageal cancer group, and the colorectal cancer group, were significantly elevated in terms of the levels of anti‐PAIP1 and anti‐SARS in sera. This implied that PAIP1 and SARS may play a role in multiple tumors and may be tumor‐associated antigens instead of HCC‐specific associated antigens. Meanwhile, it was consistent with other studies [28, 29, 30]. Descriptions of the detailed biological functions of eight TAA were summarized in Table S1 [31, 32, 33, 34, 35, 36]. On the basis of the literature, these eight TAAbs identified as biomarkers for early detection of HCC in this study were barely reported. These eight TAAbs showed the diagnostic performance for HCC, which may imply that the corresponding TAAs might play critical roles in the occurrence and development of HCC, and it is critical to explore the biological function of the TAAs in the progression of HCC.

The development of HCC is considered to be a complex multistep process, which is related to chronic inflammatory damage and liver cirrhosis [2, 4]. The sustained inflammatory process caused by chronic hepatitis B virus infection stimulates fibrosis to cirrhosis and HCC [37]. Therefore, in this study, we recruited not only HCC patients and normal control but also at‐risk liver diseases (chronic hepatitis B and liver cirrhosis) patients for evaluating the performance of the immunodiagnostic model. In the current study, during the transition of CHB to cirrhosis and early‐stage HCC, the PP value of the immunodiagnostic model increased gradually, which may be due to the immune responses to the qualitative or quantitative changes in the proteins corresponding to the six autoantibodies by the immune system. The results also indicated that the immunodiagnostic model value in this study is associated with the progression of liver fibrosis to HCC. Besides, the median of the predictive probability value of the immunodiagnostic model in patients with advanced HCC was significantly higher than that in the normal control group and at‐risk control group, but lower than that in the early‐stage HCC patients. The similar phenomenon appeared in several other scholars' researches, and the decrease of PP value in the advanced HCC may be due to the result of the loss of antigens to help the tumor escape immune [38, 39].

Besides, HCC model mice could provide samples from the latency period in the process of hepatocarcinogenesis and are suitable for evaluating novel serum biomarkers before clinical application [40]. Here, the levels of the six TAAbs were gradually increasing with time going in the serial serum samples collected from HCC model mice, whereas they appeared at relatively stable levels in control groups. The results indicated that TAAbs might be suitable biomarkers for early detection of HCC due to early appearance before the imaging could detect the tumor formation.

Alpha‐fetoprotein has been widely used in clinical diagnosis of multiple tumors, especially HCC. Therefore, one of the focuses in this study is whether TAAbs can enhance or supplement AFP as biomarkers for HCC diagnosis. The diagnostic performance of the immunodiagnostic model in this study did not show significant difference between AFP‐positive and AFP‐negative groups. The fact is that the immunodiagnostic model can distinguish 77.1% of HCC patients with AFP‐negative group, which suggests that the immunodiagnostic model can be used as a supplemental biomarker for detecting the AFP‐negative patients as the one previously identified [10]. Besides, the results hint that the combination of AFP and immunodiagnostic model could enhance the efficiency of HCC detection. In fact, when we combined AFP and PP value of the immunodiagnostic model, the diagnostic performance was better than that of either AFP or the 6‐TAAb panel. This finding was similar to that of a study in the United Kingdom [41]. The performance of the immunodiagnostic model and AFP had been validated by multiple cohorts and in both human and murine sera. However, the limitation of the study is that the serial serum samples in human from the transition of cirrhosis to HCC are currently not available. Besides, the performance of TAAbs screened from the protein encoded by cancer driver genes in a previous study didn't show the top 25 biomarkers in the Human Proteome Microarray [10]. This may be partly due to the different samples in the two studies, on the other hand the constitution rates of the samples were different. Therefore, setting up a prospective cohort study to collect the serial serum samples will be one of our further study plans.

## 5 Conclusions

Our study showed that the combination of the immunodiagnostic model by the TAAbs and AFP could enhance the HCC detection, especially for AFP‐negative HCC patients. Since the TAAbs identified in this study were observed to be elevated in the sera of mice with precancerous lesions, these TAAbs can be used as biomarkers in the early detection of HCC.

## Conflict of interest

The authors declare no conflict of interest.

## Author contributions

PW and XW provided the direction and guidance throughout the preparation of the study. QY and HY conducted the experiments, wrote and edited the manuscript. XW, PW, GYS, KYW, LPD, and CPQ revised the manuscript, and assisted in analyzing the data in this study. HY and JXS provided the guidance for solving the problems in the process of experiments. JCZ prepared the related literature search and collected the data collection of the patients' clinical information. All authors approved the final manuscript.

### Peer review

The peer review history for this article is available at https://publons.com/publon/10.1002/1878‐0261.13371.

## Supporting information


**Fig. S1.** The scatter plots of the optical density (OD) values of the TAAbs by ELISA in test set (a), training set (b), and validation set (c).
**Fig. S2.** The receiver operator characteristic (ROC) curve of the optical density (OD) values for TAAbs by ELISA in training set (a), and validation set (b).
**Table S1.** The descriptions of the 15 candidate TAAs.

## Data Availability

All the data in this study are available from the corresponding authors upon reasonable requirement.
