# Supplementary material for: Human Proteome Microarray identifies autoantibodies to tumor‐associated antigens as serological biomarkers for the diagnosis of hepatocellular carcinoma
Source: Mol Oncol. 2023 Jan 21;17(5):887–900. doi: 10.1002/1878-0261.13371 (PMC10158779; doi:10.1002/1878-0261.13371)
Supplement: Supplementary file 1 — Fig. S1. The scatter plots of the optical density (OD) values of the TAAbs by ELISA in test set (a), training set (b), and validation set (c). Fig. S2. The receiver operator characteristic (ROC) curve of the optical density (OD) values for TAAbs by ELISA in training set (a), and validation set (b). Table S1. The descriptions of the 15 candidate TAAs. [file MOL2-17-887-s001.docx]

**Supplementary Figures and Tables**

**
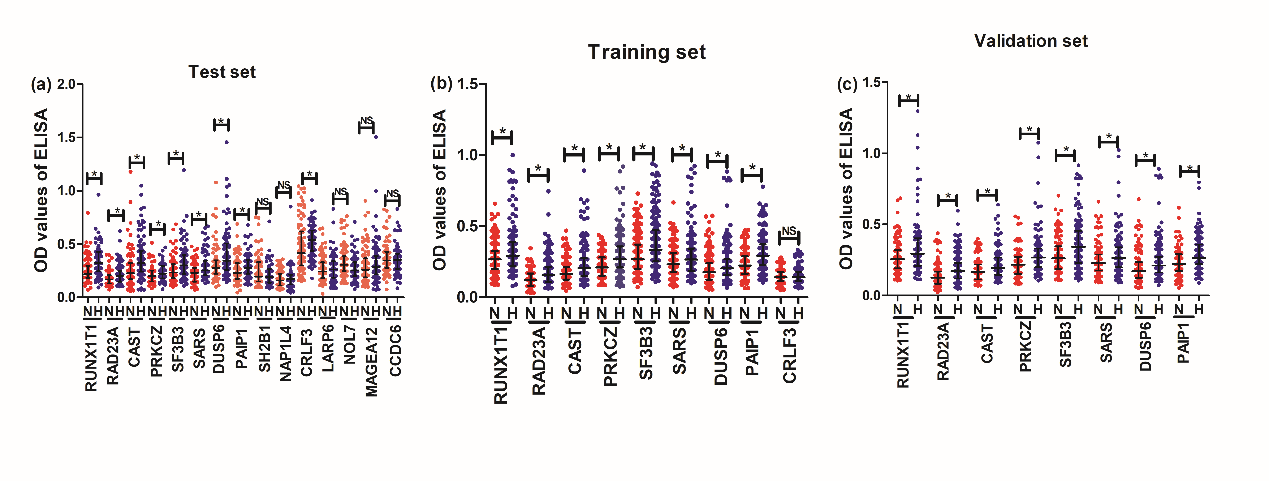
**

**Figure S1.** The scatter plots of the optical density (OD) values of the TAAbs by ELISA in test set (a), training set (b), and validation set (c). *: *P*<0.05 by the Mann-Whitney U test. ELISA, Enzyme linked immunosorbent assay; TAAb, autoantibody to tumor associated antigen; NS, no significant; H, hepatocellular carcinoma; N, normal controls. The line on scatter plots was the median with interquartile range.

**
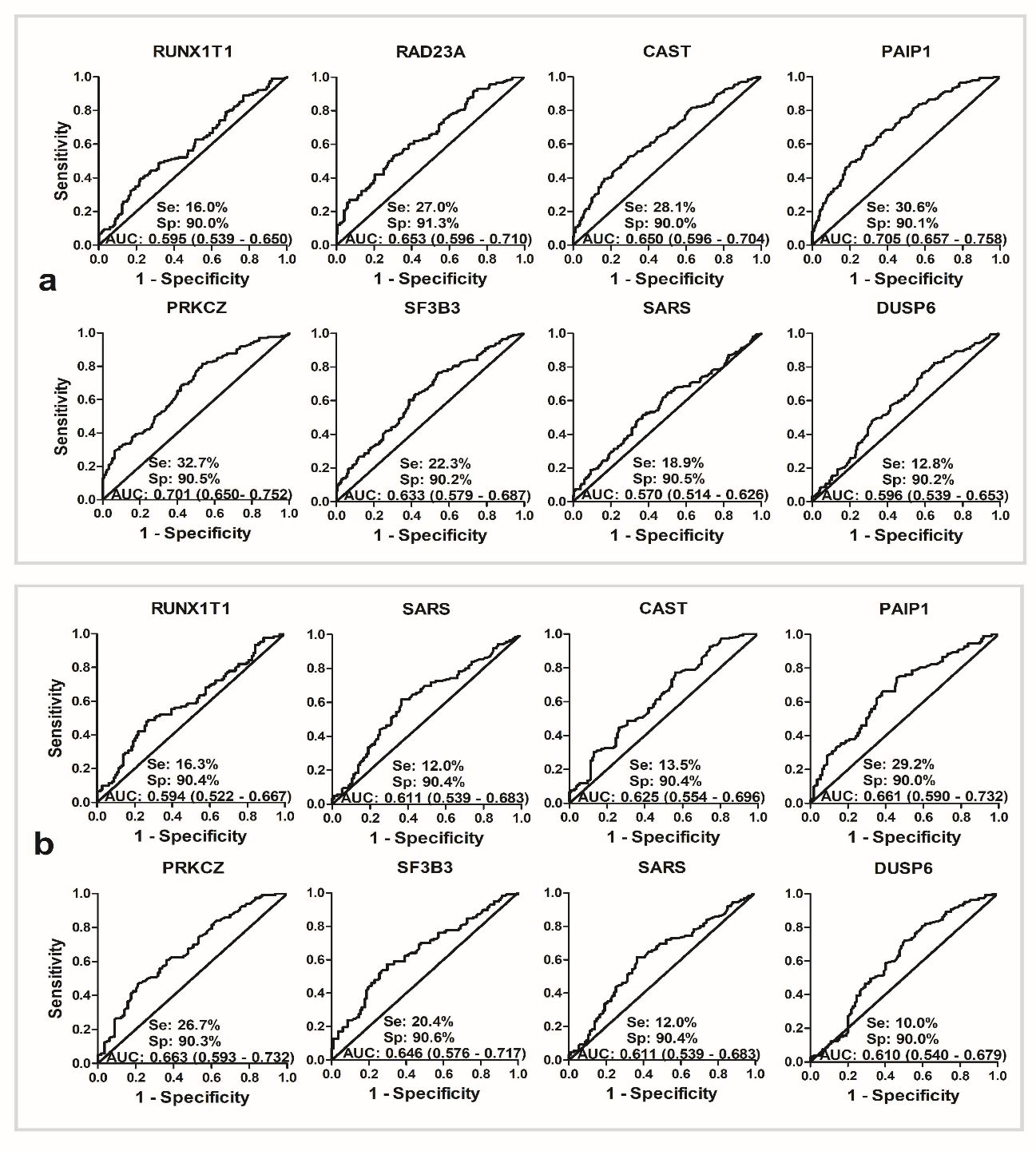
**

**Figure S2.** The receiver operator characteristic (ROC) curve of the optical density (OD) values for TAAbs by ELISA in training set (a), and validation set (b). ELISA, Enzyme linked immunosorbent assay; TAAb, autoantibody to tumor associated antigen; Se, sensitivity; Sp, specificity; AUC, area under curve.

Table S1 The descriptions of the 15 candidate TAAs

| TAAs | Gene Name | Protein name (s) | Core pathway | Function / process | Website/literature |
| --- | --- | --- | --- | --- | --- |
| CAST | CAST | Calpastatin, calpain inhibitor, sperm BS-17 component | CDK5 | Specific inhibition of calpain | https://www.proteinatlas.org/ENSG00000153113-CAST |
| CCDC6 | CCDC6, TST1, D10S170 | Coiled-coil domain-containing protein 6, Papillary thyroid carcinoma-encoded protein, Protein H4 | ------- | Oncogene | https://www.proteinatlas.org/ENSG00000108091-CCDC6 |
| CRLF3 | M9, CRLM9,  CREME9, CRLF3, P48, CYTOR4 | Cytokine receptor-like factor 3, Cytokine receptor-like molecule 9, Cytokine receptor-related protein 4, Type I cytokine receptor-like factor p48, CREME-9 | JAK-STAT | ------- | https://www.proteinatlas.org/search/CRLF3 |
| DUSP6 | DUSP6, MKP3, PYST1 | Dual specificity phosphatase 6, MAP kinase phosphatase 3, MKP-3 | MAPK1/MAPK3 | Inactivates MAP kinases | https://www.proteinatlas.org/ENSG00000139318-DUSP6 |
| LARP6 | LARP6 | La-related protein 6, Acheron, Achn, La ribonucleoprotein domain family member 6 | Translation regulation, | RNA-binding | https://www.proteinatlas.org/ENSG00000166173-LARP6 |
| MAGEA12 | MAGEA12, MAGE12 | Melanoma-associated antigen 12, Cancer/testis antigen 1.12, MAGE-12 antigen, MAGE12F antigen, CT1.12 | Tumor antigen | Not known, may play a role tumor transformation or progression | https://www.proteinatlas.org/ENSG00000213401-MAGEA12 |
| NAP1L4 | NAP1L4, NAP2 | Nucleosome assembly protein 1-like 4, Nucleosome assembly protein 2, NAP-2 | --------- | histone chaperone in nucleosome assembly | https://www.proteinatlas.org/ENSG00000205531-NAP1L4 |
| NOL7 | NOL7, C6orf90, NOP27 | Nucleolar protein 7, Nucleolar protein of 27 kDa | --------- | RNA-binding | https://www.proteinatlas.org/ENSG00000225921-NOL7 |
| PAIP1 | PAIP1 | Polyadenylate-binding protein-interacting protein 1, Poly(A)-binding protein-interacting protein 1, PABP-interacting protein 1, PAIP-1, | Deadenylation of mRNA | A coactivator in the regulation of translation initiation of poly(A)-containing mRNAs, Translation regulation | https://www.proteinatlas.org/ENSG00000172239-PAIP1 |
| PRKCZ | Protein kinase C zeta | Protein kinase C zeta type, nPKC-zeta | PI3K pathway, MAPK cascade | Kinase, Serine/threonine-protein kinase, Transferase | https://www.proteinatlas.org/ENSG00000067606-PRKCZ |
| RAD23A | RAD23A | UV excision repair protein RAD23 homolog A, HR23A, hHR23A | P53 degradation | DNA damage, DNA repair, Host-virus interaction | https://www.proteinatlas.org/ENSG00000179262-RAD23A |
| RUNX1T1 | RUNX1T1, ETO, AML1T1, MTG8, CDR, CBFA2T1, ZMYND2 | Protein CBFA2T1, Cyclin-D-related protein, Eight twenty one protein, Protein ETO, Protein MTG8, Zinc finger MYND domain-containing protein 2 | Transcription regulation | DNA-binding, Transcriptional corepressor activity | https://www.proteinatlas.org/ENSG00000079102-RUNX1T1 |
| SARS | SARS1, SARS, SERS | Serine--tRNA ligase, cytoplasmic, SerRS, Seryl-tRNA(Ser/Sec) synthetase | Selenocysteinyl-tRNA(Sec) biosynthesis | Aminoacyl-tRNA synthetase, DNA-binding, Ligase | breast cancer^1^ |
| SH2B1 | KIAA1299 | SH2B adapter protein 1, Pro-rich, PH and SH2 domain-containing signaling mediator, SH2 domain-containing protein 1B, PSM | Janus kinase (JAK) and receptor tyrosine kinases | Transmembrane receptor protein tyrosine kinase adaptor activity | https://www.proteinatlas.org/ENSG00000178188-SH2B1 |
| SF3B3 | SF3B3,  SAP130,  KIAA0017 | Splicing factor 3B subunit 3, SAP 130, Pre-mRNA-splicing factor SF3b 130 kDa subunit, SF3b130, STAF130 | mRNA Splicing | Involved in pre-mRNA splicing as a component of the splicing factor SF3B complex | breast carcinoma^2^ |

**References:**

1. Zhao J, Bai H, Li X, et al. Glucose-sensitive acetylation of Seryl tRNA synthetase regulates lipid synthesis in breast cancer. Signal Transduct Target Ther 2021*;* **6**(1): 303*.*[PubMed: 34400610]

2. Zhang S, Zhang J, An Y, et al. Multi-omics approaches identify SF3B3 and SIRT3 as candidate autophagic regulators and druggable targets in invasive breast carcinoma. ACTA PHARM SIN B 2021*;* **11**(5): 1227-1245*.*[PubMed: 34094830]
